# Spatial Effects of Technological Progress and Financial Support on China’s Provincial Carbon Emissions

**DOI:** 10.3390/ijerph16101743

**Published:** 2019-05-16

**Authors:** Yingying Zhou, Yaru Xu, Chuanzhe Liu, Zhuoqing Fang, Jiayi Guo

**Affiliations:** School of Management, China University of Mining and Technology, Xuzhou 221116, Jiangsu, China; zhouyingying@cumt.edu.cn (Y.Z.); zyy98yafa@126.com (Y.X.); zgh60wse@126.com (Z.F.); yijiaguoyaya@163.com (J.G.)

**Keywords:** technical progress, financial support, carbon intensity, spatial autocorrelation

## Abstract

The spatial autocorrelation analysis method was applied to panel data from the provinces of China (including autonomous regions and municipalities directly under the central government) for the period 2003 to 2016 in order to construct a spatial Durbin model of technological progress and financial support in relation to reductions in carbon emissions. The results show that China’s carbon intensity presents significant spatial spillover effects under different spatial weights, which indicates that the carbon intensity of a province is influenced not only by its own characteristics, but also by the carbon emission behaviors of geographically adjacent and economically similar provinces and regions. Financial structure, financial scale, and financial efficiency all have significant effects on carbon intensity within a province, while financial structure is also linked to carbon intensity in other regions, but financial scale has no significant spillover effect on carbon intensity in space. Areas with high financial efficiency can reduce their own carbon intensity as well as that of surrounding areas. The inter-regional spillover effect of technological progress on carbon intensity is stronger than the spillover effect, but there is a time lag.

## 1. Introduction

The report of the 19th National Congress of the Communist Party of China placed “ecological civilization construction” high on the national strategic agenda, and governments and enterprises at all levels have attached increasing importance to improving production technology, developing renewable energy, and establishing and perfecting a national carbon emissions trading market to promote emissions reduction. Under the 2015 Paris Agreement on climate change, China proposed that its carbon dioxide emissions per unit GDP would be reduced by 60–65% by 2030 compared with 2005. Although China has made great efforts to reduce carbon emissions, its economic development has meant that energy consumption has surged, and total emissions have remained high. According to the International Energy Agency, China’s carbon emissions in 2016 totaled 9.123 billion tons, more than any other country in the world. Carbon emissions may become a factor that restricts the rapid and healthy development of China’s economy. To reduce carbon emissions at the same time as ensuring sustainable economic growth, it is necessary to change the country’s original development model, which was highly dependent on carbon energy.

There is a close relationship between carbon emissions, environment, and public health. Chinese President Jinping Xi pointed out at the 19th National Congress of the Communist Party of China that in order to meet the growing needs of the people for a beautiful ecological environment, we should strengthen the relationship between the construction of an ecological civilization in China and its participation in the global response to climate change; moreover, we should attach great importance to solving serious environmental problems that damage human health. We should insist on giving priority to pollution prevention in relation to air, water, and soil. China should take the initiative to strengthen the construction of an ecological civilization, control carbon emissions, implement emission reduction commitments, and provide more high-quality ecological products and a higher-quality ecological environment. Sustainable development will contribute to global ecological security.

Emission reductions technology and energy efficiency technology are improving China’s energy efficiency and changing China’s high-carbon industrial structure. For example, waste incineration power generation can contribute 2–10% of China’s electricity output while realizing a carbon emissions reduction of ~7.65 million tons. From 2006 to 2015, oxygen-enriched carbon combustion, more efficient energy processing and conversion, and power generation from renewable sources achieved carbon emissions reductions of approximately 1.345 billion tons, 2.44 billion tons, and 8.5 billion tons, respectively. Other measures have included reducing or even eliminating some industries with high energy and water consumption, as well as highly polluting industries and encouraging enterprises to introduce energy-saving and emission reduction technologies.

The Ministry of Science and Technology, the Ministry of Environmental Protection, and the Meteorological Bureau of China have jointly issued the “13th Five-Year Plan for Scientific and Technological Innovation to Address Climate Change”, which states that, of the major global economies, China is also the country with the greatest pressure for economic transformation. Since reform and opening up, China’s economic and social development have been remarkable. However, in general, since China is still in the process of industrialization and urbanization, the proportion of secondary industry is relatively large, and coal is still the main energy source, resulting in high energy consumption per unit GDP and large greenhouse gas emissions. This mode of development and energy consumption has worsened the atmospheric environment, endangered people’s lives and health, and even threatened national security. There is an urgent need to switch to low-carbon development. In this respect, it is important to assess the impact of financial policies (such as green credit and other financial mechanisms) on China’s transformation to a low-carbon economy, and to study the effect of market-based environmental energy and climate change policies on, for example, carbon emissions, air pollution, water resources, water pollution, and human health.

In March 2018, China’s financial assets totaled ~247.95 trillion yuan, and the balance of domestic and foreign currency loans was 130.45 trillion yuan, up 11.9% compared with the previous year. The balance of domestic and foreign currency deposits was 174.44 trillion yuan, up 8.4% year-on-year. China has realized the importance of technological innovation. At the end of 2017, the amount of patent pledge financing in China exceeded 72 billion yuan, and the capital raised by the patent operation fund of key industries was 4.28 billion yuan. In 2017, the national average increase in R&D investment was 11% and the number of patent applications reached 1.382 million, an increase of 14.2% over the previous year. The government promotes companies with interests in (and owning intellectual property for) core advanced technologies. Financial support, when guided by the optimal allocation of resources, plays an important part in driving economic development. Reducing carbon emissions cannot be separated from financial support, and a higher level of financial development is conducive to promoting technological progress, which in turn plays an important role in improving energy efficiency and reducing carbon emissions.

In the report on the work of the Chinese government in 2019, Premier Keqiang Li pointed out that China’s stage of development offers strategic opportunities for people to pursue a better life, with sufficient resilience, great potential, and innovative vitality. They have the firm will and ability to overcome all kinds of difficult challenges, and the long-term trend of economic improvement has not changed, nor will change. The main target for Chinese economic development in 2019 is to increase GDP by 6–6.5%. More than 11 million people are employed in cities and towns. Such growth in recent years has been achieved alongside improvements in the ecological environment. For example, energy consumption per unit GDP has dropped by ~3% and the emission of major pollutants has continued to decline. Indeed, China’s economic development has been successfully linked to the goal of building a well-off society in a rounded, positive, and stable way.

In recent times the financial development model has gradually shifted to a carbon finance model. China’s total green financing amounted to approximately 9 trillion yuan in 2017. The liquidity of the carbon emissions trading market has gradually increased through innovations in green bonds, green funds, and financial derivative products, which in turn has led to greater energy conservation and emission reductions, better environmental governance, and economic restructuring. It is therefore of great practical significance to study the spatial characteristics of carbon emissions in China’s provinces (including autonomous regions and municipalities directly under the central government) and to explore the influence of financial development and technological progress on carbon emissions.

## 2. Literature Review

Economic development is almost invariably accompanied by the problems of high energy consumption, high pollution, high emissions, and so on, and these have exerted great pressure on China. The contradiction between China’s economic development (and associated energy consumption) and its environmental well-being is becoming increasingly prominent. Nonetheless, it has been proposed that reducing carbon emissions may be a way to alleviate both economic and environmental problems (Dan Tan, 2008 [1]; Xue Leng, 2012 [2]; Ling Wang, 2016 [3]). Domestic and foreign scholars have assessed the effects of carbon emission reduction using models that incorporate economic growth, the openness of trade, financial support, resource allocation, technological progress, carbon reduction efficiency, population structure, and other indicators. The results emphasize the importance of financial support and technological progress.

### 2.1. Factors Affecting Carbon Emissions: Openness of Trade, Population Size, Environment, Human Capital, Output Per Capita, Industrial Structure, Gross Output, Energy Structure, and Energy iIntensity

Kai Li and Shaozhou Qi (2011) [4] used the panel data from 30 provinces (including autonomous regions and municipalities directly under the central government) over the period 1997 to 2008 to research the environmental effects of international trade based on the theories related to free trade opening, economic growth, and carbon emissions. They found that the international trade has a negative impact on the quality of China’s environmental quality of our country.

Bassem Kahouli (2017) [5] analyzed the panel data for countries in the South Mediterranean countries from 1995 to 2015. They established an autoregressive distribution lag model and a vector error correction model that took taking trade, capital, and urbanization as controlled variables to examine the long-term and short-term causal relationships among economic growth, energy consumption, and financial development. It was found that there was a single effect among the three variables in most countries. They proposed that an increase in the level of financial development would reduce unreasonable energy consumption and carbon dioxide emissions and protect the environment while the economy continued to grow.

Yang Yu, Feifan Chen (2017) [6] used the data from China and Korea over the period 2000 to 2010 to establish an input–output model to examine the relationship between trade and carbon emissions. The results showed that trade transfer between China and South Korea played a positive role in reducing global carbon emission, and that the textile, leather, chemical, and metal markets were the main areas responsible for increasing carbon emissions. Bing Sun (2011) [7] used Sichuan panel data from 2000 to 2009 to examine the effects of population, and economic and technical factors on carbon emissions. Sichuan Province is a low-carbon economy. The results showed that the population size had a greater impact on the carbon emission than the population structure, and the authors proposed controlling population growth, advocating a low-carbon, low-consumption lifestyle.

### 2.2. Energy Consumption

Energy shortage and environmental deterioration have attracted the attention of the international community, and in particular the problem of climate change. The optimization of energy structure, and especially the reduction of energy intensity, has contributed to a reduction in carbon emissions (Wang, 2005 [8]; Chang, 2008 [9]; Ugur, Ramzan, 2009 [10]; Ohanlan R, 2015 [11]). Magazzino (2015) [12] believed that overconsumption of energy was the cause of increases in carbon emissions. Freitas and Kaneko (2011) [13] found that a reduction in carbon intensity and the optimization of energy structure contributed to reductions in carbon emissions.

Shiyi Chen (2009) [14] discussed the sustainable development of those parts of China’s industry characterized by high energy consumption and high carbon emissions, and found that energy and capital were the main factors driving China’s industrial growth, and that fully sustainable development of China’s industry would require improvements to energy-saving and emission reduction technologies.

Sadia Bano, Yuhuan Zhao, and Ashfaq Ahmad et al. (2018) [15] used panel data from Pakistan over the period 1971 to 2014 and established an autoregressive distribution lag model and a vector error correction model to analyze the relationship between human capital and carbon emissions. The study showed that the improvement of human capital would reduce carbon emissions, but would not restrain economic growth. Yuanming Ou and Shaofu Zhou (2015) [16] used the STIRPAT model to study the factors associated with per capita carbon emissions in China and found that per capita output, foreign trade, energy intensity, energy structure, and other factors led to an increase in per capita carbon emissions, while population density, industrial structure, and urbanization rate could curb carbon emissions.

Wanbo Lu, Tingting Qiu, and Lei Du (2013) [17] studied the contribution of the six major industrial sectors to carbon emissions from the perspective of industrial structure, and also considered the effects of energy structure, energy intensity, industrial structure, and gross output value on carbon emissions. It was found that gross output value and industrial structure contributed to carbon emissions, while energy intensity and energy structure could restrain increases in carbon emissions.

### 2.3. The Impact of Transportation, Highway Construction, Construction, and Tourism Development on Carbon Emissions

Xiaoli Lu and Xiaofei Lv (2015) [18] analyzed the impact of transportation on carbon emissions in Liaoning Province by using the Sharp decomposition method to, and found that increasing the utilization rate of transportation could reduce the carbon emissions caused by transportation. Rui Xie, Jiayu Fang, and Cenjie Liu (2017) [19] established a STRIPAT model to study the impact of transport infrastructure on carbon emissions in cities based on the panel data from 283 cities in China over the period 2003 to 2013.The results showed that compared with small cities, transport infrastructure has led to a more significant increase in carbon emissions in large and medium-sized cities. Bo Peng, Xueyong Fan, and Xunjie Wang (2017) [20] found that asphalt heating and aggregate drying in highway construction were the major causes of carbon emissions in many provinces of China. Kai Wang, Haiqin Shao, and Tingting Zhou et al. (2018) [21] used the extended environmental Kuznets curve (EKC) to study the impact of tourism development on regional carbon emissions, their data analysis showed that the development of tourism would lead to an increase in carbon emissions but the professional level of tourism services could reduce the environmental pressure of economic development.

### 2.4. The Impacts of Financial Support and Technological Progress on Carbon Emissions

Finance is the core of the modern economy, and it plays an important role in promoting environmental protection and in transforming of the mode of economic development. The empirical results from Qian Wang, Xing Shuang, and Rui Huang (2012) [22]; Hongmei Gu and Bin He (2012) [23]; Wanli Zhu (2015) [24]; and Liya Hao (2016) [25], among others, confirmed that financial support played a significant role in the suppression of carbon emissions, and financial development is conducive to the development of a low-carbon economy.

Hong Jiang (2018) [26] found that improvements in financial scale and financial efficiency have significantly improved regional energy efficiency. Mei Song (2016) [27] found that financial development in a central region would lead to a reduction in carbon emissions. Zhang (2011) [28] built an econometric model based on China’s national conditions. The empirical results show that financial development can not only promote economic growth, but also indirectly restrain carbon emissions by stimulating technological innovation. Li (2019) [29] believes that the development of green credit will help reduce carbon emissions.

Ibrahim (2018) [30] found that international trade and financial development have complementary effects in reducing carbon emission intensity; that is, the more mature the financial system is, the more obvious the environmental benefits of international trade will be; similarly, under highly open economic conditions, financial development is conducive to reducing carbon emission intensity.

However, Junjun Guo, Chengyu Liu, and Yuping Liu (2012) [31]; Biqiong Chen and Liangliang Zhang (2014) [32]; and Ling Xiong and Shaozhou Qi (2016) [33] had opposite results in their empirical analysis. They found that an improvement in the level of financial development could promote economic growth, which in turn would increase the demand for energy, and energy consumption would inevitably lead to an increase in carbon emissions inevitably.

According to Lihua Zhang, Jiali Ren, R.Wang (2017) [34], and others, the development of the finance industry can increase carbon dioxide emissions, but the development of the insurance industry and innovation in insurance products can help reduce carbon emissions.

In a study on the relationship the economy and the environment, Shafik (1992) [35] proposed that when the tertiary industry was the leading sector, the economy would develop further, the demand for capital and technology would increase, the demand for energy would be reduced, the efficiency of technology and the energy use efficiency would be further improved, and carbon emissions would be reduced, supporting an improvement in environmental quality. Ling Xiong and Shaozhou Qi (2016) [33] believe that financial development can not only increase carbon emissions by exerting a scale effect (expanding economic aggregates and enterprise productivity) and a wealth effect (increasing consumer income and borrowing, which in turn expand the purchase of energy-consuming commodities), but also restrain carbon emissions by a structural effect (increasing the proportion of tertiary industry) and a technological effect (accelerating technological progress of enterprises). However, their empirical results show that financial development has a positive effect on carbon emissions, and the structural effect and technological effect have not been fully developed in China.

Di Wang, Rui Nie (2010) [36], Kegui Zuo, Yuze Bao et al. (2014) [37], Jianlan Ren (2015) [38], and Xilong Yao (2013) [39] also confirmed that technological progress can play a leading role in reducing carbon emissions. Bing Wang and Minzhe Du (2015) [40] believed that low-carbon technology could both realize both a reduction in carbon emissions and an increase in industrial output. Based on the production theory, the DEA model of carbon emission reduction efficiency was established, and the factors affecting the efficiency of carbon emission reduction efficiency were decomposed. Wei Zhang, Qigui Zhu, and Hanwen Li (2013) [41] found that the technology of carbon emission reduction and energy utilization had a strong positive impact on the efficiency on carbon emission reduction efficiency, and they proposed improvements in energy utilization and carbon emission reduction technology. Wensong Su, Yanyan Liu, and Shaojian Wang et al. (2018) [42] used spatial correlation analysis, a Markov chain transition matrix, a dynamic panel model, and generalized moment estimation to empirically analyze the influencing factors influencing urban carbon emissions in 19 countries. The factors such as population aggregation, technological progress, and trade openness all restrained carbon emissions. Optimizing the industrial structure, simplifying investment, and improving the level of technology were the most effective ways to save energy and reduce emissions. Kaijie Li and Ruxiao Qu (2012) [43] considered that technological progress could reduce carbon emissions in the longterm, but in the short term technological progress had no obvious effect on carbon emissions. Hongguang Liu, Weidong Liu, and Fan Xiaomei (2011) [44] believed that the adjustment of industry structure and technological progress had little effect on carbon emission reduction in China.

With regard to researches on the promotion of technological innovation due to financial development to promote carbon emission reduction, Linares and Perez-Ariaga (2009) [45] and Gouvello (2010) [46] found that by improving the level of financial development of a country or region, it was possible to aggregate a large amount of slack funds in society to support the progress in low-carbon technology and to invest in the development of “new” (green) energy, which could help to reduce carbon dioxide emissions. The results of Beck, Levine, and Loayza (2000) [47] and Hanson and Laitner (2004) [48] showed that the financial development through the promotion of technological innovation could improve the full-factor productivity of the economy and contribute to reductions in carbon emissions. Fuente and Marín (1995) [49], Tamazian, Chousa, and Vadlamannati (2009) [50] believed the function of financial resource allocation could guide funds to support for the development of low-carbon technology to improve the efficiency of energy utilization, alleviate the pressure of energy shortage, reduce environmental pollution, and promote the development of a low-carbon economy. Based on the theoretical model of an inverse U-shaped relationship between financial development and carbon dioxide emissions intensity, Chengliang Yan, Tao Li, and Wei Lan (2016) [51] used panel data from 30 provinces (including autonomous regions and municipalities directly under the central government) in China over the period 1997 to 2012 to construct an endogenous growth model that included financial development, innovation and carbon dioxide intensity, as well as other variables. They put forward the view that financial development could not only directly promote the development of a low-carbon economy not only directly, but also indirectly promote low-carbon economy through technological innovation and other channels.

### 2.5. Conclusions from the Literature Review

It can be seen from the above analysis that both domestic and foreign scholars have produced some valuable research results on the factors that impact carbon emissions in China. However, the research needs to be extended in two key respects. Firstly, most studies have used financial scale as the indicator for financial development, whereas in fact financial development is reflected not only in the expansion of financial scale, but also in the improvement of both financial structure and financial efficiency. Secondly, existing studies neglect the spatial correlation and spillover effects of financial development and technological progress on carbon emissions, and so fail to truly reflect the relationship between financial development, technological progress, and carbon emissions. Therefore, the present study draws on the research experience of domestic and foreign scholars on the factors that influence carbon emissions. It uses empirical data on economic development in China, taking the provinces, autonomous regions, and municipalities directly under the central government as the research object, using provincial panel data from 2003 to 2016, to analyze the impact (in terms of both direction and degree) of financial support and technological progress on the intensity of carbon emissions in spatial (regional/provincial) terms. An exploration of the relationship between financial support, technological progress, and carbon dioxide emissions is of great significance for the promotion of sustainable and healthy economic development.

## 3. Model Setting and Data Description

### 3.1. Model Setting

Levine (2004) [52] proposed five main functions of financial system based on the theory of financial function: Firstly, the function of resource allocation; secondly, the function of risk control; thirdly, the function of promoting the flow of social capital; fourthly, the function of promoting the circulation of goods and services; and fifthly, the function of promoting the optimization of enterprise management structure. Financial functionalism promotes the enrichment and development of financial development theory, which shows that there is a close relationship between financial development and economic growth.

**Figure 1:**
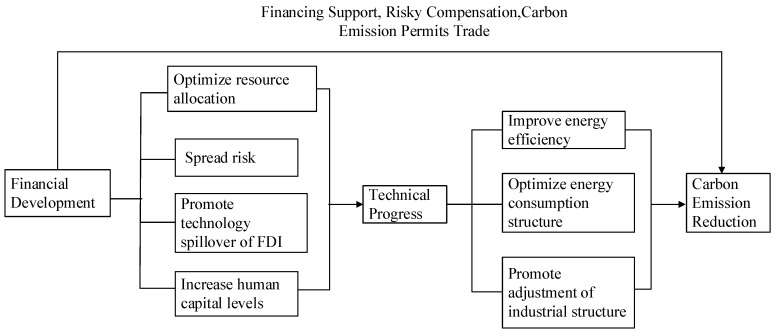
Path analysis of financial development affecting carbon emission reduction.

Chengliang Yan, Tao Li, and others (2016) [51] proposed that financial development cannot only directly affect carbon emission reduction, but also indirectly do so through factors such as technological progress. At the end of 2017, carbon emissions per unit GDP in China had decreased by 46% compared with 2005. Dr. Xian Zhang of the Department of 21st Century Center Global Environment reported that technological progress was responsible for ~60% of this decrease. That is, technological progress was the main factor driving carbon emission reduction. Research has indeed shown that technological progress plays a very important role in the relationship between financial development and carbon emission reduction. For example, Birdsall and Wheelerr (1993) [53] believed that higher levels of financial development are more conducive to attracting FDI and high-level R&D investment, which in turn promote technological progress, which, as set out above, can increase the quality of the environment. Developing countries should therefore provide incentives and opportunities for the use of new technologies and equipment, and thereby promote the production of more environmentally friendly products. Muhammad Shahbaz, Sakiru Adebola Solarin, Haider Mahmood et al. (2013) [54] believe that financial development can promote technological research and development and innovation by reducing financing costs and dispersing risks, and that this technological progress can help energy production sectors reduce their carbon dioxide emissions; moreover, financial development can create a good financial environment and attract FDI, which will help host countries to improve their technology and so reduce their carbon emissions. Therefore, this paper further analyzes how financial development affects technological progress and promotes carbon emission reduction, as shown in Figure 1.

Technology R&D has high financing cost. Firstly, the achievements of technological research and development are unknown. Moral hazards caused by information asymmetry often occur in the process of technological research and development, which makes the process of technological progress lack of necessary external financing. Secondly, the road of transforming knowledge into products is very difficult and long. High investment, high risk, and long cycle in the process of technological progress make financing difficult, and it is an important problem in the process of technological research and development. These characteristics show that technological progress depends on long-term investment, and it is difficult to regain costs in the short term. Therefore, sustained and stable financing is the necessary support for technological research and development.

The resource allocation function of the financial system can arrange the limited resources in various fields rationally, so that all kinds of resources in society can be allocated to better meet social needs effectively. As a bridge between the supplier and the demander of funds, the financial system has an efficient savings and investment transformation mechanism. It can greatly improve the utilization rate of funds and promote technological progress by promoting resource allocation by gathering idle funds in the field of social circulation, and then choosing high-quality projects for investment. Compared with individual investors, financial intermediaries can achieve scale effect, disperse the cost of information acquisition among many investors, reduce the cost of information acquisition units effectively, have a professional team that can specialize in information processing, and select projects and enterprises with investment value, so as to promote the flow of resources to high-efficiency areas.

On the impact of technological progress on energy efficiency, Fisher Vanden et al. (2004) [55], Li Lianshui and Zhou Yong (2006) [56], Zhu Yanfu and Teng Yuhua (2010) [57], and Pan Xiongfeng and Peng Xiaoxue (2017) [58] found that technological progress is an important driving force for improving energy efficiency. Dong Feng, Tan Qingmei, and Zhou Dequn (2010) [59] used panel data to analyze the impact of technological progress on energy efficiency. The results show that technological progress contributes greatly to energy efficiency. In terms of energy consumption, increasing R&D expenditure will provide more financial support for R&D projects related to energy technology, energy utilization equipment and management, so as to improve the level of product or engineering design and development, or update equipment technology, improve the service life of equipment systems, thereby reducing energy consumption in production process and improving energy utilization efficiency. However, the continuous accumulation of human capital can improve energy utilization equipment, optimize the allocation of other factors of production, raise awareness of energy conservation, promote the transformation of energy consumption mode, change from extensive energy consumption to intensive energy consumption, and further improve energy utilization.

The path by which financial development affects technological progress is as follows. Firstly, financial development can realize the effective use of capital resources by giving priority to the allocation of limited resources to projects and enterprises advancing technological progress. Secondly, the higher the level of financial development is, the stronger is its ability to diffuse risks. Thirdly, an increase in the level of financial development (i.e., a good financial environment) will attract more foreign investment, and foreign direct investment cannot only promote local economic development, but also make technological innovation (technological progress) less costly. Finally, financial development can finance the accumulation of as well as the level of human capital.

The path by which technological progress affects carbon emissions is as follows. Firstly, technological progress can increase energy efficiency. With more advanced carbon emission reduction technology and the popularization of low-carbon equipment and machinery, the energy utilization rate will be improved, thus promoting reductions in carbon emissions. Secondly, technological progress can improve the structure of energy consumption. For instance, technological progress will lead to many sources of renewable energy being developed and utilized. Already, the proportion of “low carbon” energy consumed has gradually increased, while the proportion of “high carbon” energy consumed has gradually decreased, so as to optimize the structure of energy consumption and reduce carbon dioxide emissions. Thirdly, technological progress can promote the optimization and upgrading of industrial structure; that is, it can reduce the proportion of high-energy industries, promote the development of low-carbon industries and emerging industries, and thereby promote reductions in carbon emissions.

As financial support and technological progress are linked to geographical location (Chengliang Yan and Tao Li, 2016) [51], the construction of the model should pay attention to the regional heterogeneity in the levels of both financial and technological development. China’s social development is also uneven. Regional financial and technological development are dynamic, progressive, cumulative processes, and are related not only to the local economic, political, and social environment, but also to the financial and technological development of adjacent areas. Moreover, there are large differences in financial and technological development not only between regions but also within regions (Zheng Shaozhi and Huang Mengyun, 2015 [60]; Wang Keliang, Meng Xiangrui, and Yang Li et al., 2015 [61]).

In order to reveal the influence of financial support and technological progress on carbon intensity, the model is preliminarily set as follows

*CO*_2_/*GDP_it_* = *α_K_X_itk_* + *μ_i_* + *ν_t_* + *ε_it_* (1)

In formula (1), *i* represents region and *t* the year; CO_2_/GDP is carbon intensity; *α_K_* is the estimated coefficient of explanatory variable *k*; *μ_i_* is the region fixed effect; *ν_t_* is the time-fixed effect; *ε_it_* is the random perturbation term; and *X_itk_* includes variables such as financial support and technological progress.

Because financial support, technological progress and carbon intensity have spatial relations and correlations among neighboring cells, it is necessary to analyze how financial support and technological progress affect regional carbon intensity and changes in carbon intensity among regions by using a spatial econometric model. The spatial Durbin model (SDM) for financial support and technological progress affecting carbon intensity is as follows

(2) $$CO_{2}/GDP_{it}=\rho W_{ij}^{m}CO_{2}/GDP+\alpha_{K}X_{itk}+\beta_{K}W_{ij}^{m}X_{itk}+\mu_{i}+\nu_{t}+\varepsilon_{it}$$

In formula (2), ρ is the spatial lag coefficient, which reflects the direction and degree of influence of carbon intensity of neighboring regions on the observed values of carbon intensity in the region of interest; $\beta_{k}$ is the estimation coefficient of the spatial lag term of the explanatory variable; *W^m^_ij_* is a spatial weight matrix to measure the geographic proximity of regions and their similarity in economic activities; *m* denotes the type of weight (*m* = 1–3, as there are three types of weight matrix, as explained below); N denotes the number of cross-sectional samples (30 provinces, autonomous regions, and municipalities directly under the central government); and T denotes the limit of cross-sectional samples (2003–2016); and the rest of the parameters are as in formula (1).

Because the parameters in formula (2) have different dimensions, it is impossible to compare and judge the influence of financial support and technological progress on carbon emissions in regions at different levels of economic development. In order to make the economic significance of each parameter clearer, a double logarithmic model, which is not influenced by dimensions, is adopted. The double logarithmic model is as follows

(3) $$\ln CO_{2}/GDP_{it}=\rho W_{ij}^{m}\ln CO_{2}/GDP+\alpha_{K}\ln X_{itk}+\beta_{K}W_{ij}^{m}\ln X_{itk}+\mu_{i}+\nu_{t}+\varepsilon_{it}$$

In addition, when any parameter in formula (3) is significantly 0 or not, different models can be evolved. When the ordinary least squares (OLS) regression model can be evolved:

(4) $$\ln CO_{2}/GDP_{it}=\alpha_{K}\ln X_{itk}+\mu_{i}+\nu_{t}+\varepsilon_{it}$$

When $\rho =0,\beta_{k}=0$, let $\varepsilon_{it}=\lambda W_{ij}^{m}\varepsilon_{it}+\gamma_{it}$, and the spatial effects between individuals are reflected in the model disturbance term, and a spatial error model (SEM) can be evolved:

(5) $$\ln CO_{2}/GDP_{it}=\alpha_{K}\ln X_{itk}+\mu_{i}+\nu_{t}+\varepsilon_{it},\varepsilon_{it}=\lambda W_{ij}^{m}\varepsilon_{it}+\gamma_{it}$$

When $\beta_{k}=0$, a spatial lag model (SLM) can be evolved, also called a spatial autoregressive model (SAR):

(6) $$\ln CO_{2}/GDP_{it}=\rho W_{ij}^{m}\ln CO_{2}/GDP+\alpha_{K}\ln X_{itk}+\mu_{i}+\nu_{t}+\varepsilon_{it}$$

In formulas (3)–(6), λ represents the spatial autocorrelation coefficient of the error term, $W_{ij}^{m}\varepsilon_{it}$ is the interaction between different unit interference items, $\gamma_{it}$ is a random impact item; $W_{ij}^{1}=\left\{ \begin{array}{l} 1,\mathrm{when}\;i\;\mathrm{and}\;j\;\mathrm{are}\;\mathrm{adjacent}\\ 0,\mathrm{Other}\;\mathrm{situations} \end{array}\right.$; $W_{ij}^{2}=\left\{ \begin{array}{l} \frac{1}{D_{ij}^{2}},\mathrm{when}\;i\;\mathrm{and}\;j\;\mathrm{are}\;\mathrm{adjacent}\\ 0,\mathrm{Other}\;\mathrm{situations} \end{array}\right.$, and *D_ij_* calculates the spherical geographical distance between area *i* and area *j* based on latitude and longitude data of regional administrative centers.

$W_{ij}^{3}=\left\{ \begin{array}{l} \frac{1}{|GDP_{i}-GDP_{j}|},\mathrm{when}\;i\;\mathrm{and}\;j\;\mathrm{are}\;\mathrm{adjacent}\\ 0,\mathrm{Other}\;\mathrm{situations} \end{array}\right.$, *GDP_i_* and *GDP_j_* represent the per capita GDP of region *i* and region *j*, respectively. *W^2^_ij_* is a 0–1 matrix according to the rook neighboring principle, the weights in which are based on whether each province or city is geographically adjacent to another, as this is more suitable for analyzing the political, cultural, and other space-dependent issues. Since Hainan Province is an island, its single neighbor is set to the nearest province, namely Guangdong. *W^2^_ij_* is a geographical weight matrix, and the inter-regional dependence and radiation effects are proportional to the geographical distance. Generally, the weights are set according to the distance calculated using the latitude and longitude of the capital city of each region. *W^3^_ij_* is an economic weight matrix, which is mainly applicable to the problem of spatial radiation effects between regions due to economic differences. In the specific estimation process, the three types of spatial weight matrix are standardized to ensure that the sum of the elements in each row of the matrix is equal to one.

### 3.2. Variable Selection and Data Description

The research sample comprises 30 provinces (including autonomous regions and municipalities directly under the central government) in China, excluding Tibet, Hong Kong, Macao, and Taiwan. Data were missing for the Tibet Autonomous Region in some years in the “China Statistical Yearbook” (entries were left blank). Hong Kong and Macao are special administrative regions of China, and Taiwan is relatively independent, and so the “China Statistical Yearbook” does not include statistical data on these three regions. For these reasons, the four regions of Tibet, Hong Kong, Macao, and Taiwan are excluded from the empirical analysis. The time span is from 2003 to 2016. The data set comprises the following.

The index of carbon emissions used here is carbon intensity, denoted Y, where Y = CO_2_/*GDP*, in tons/10,000 yuan. The *GDP* data for each region for each year are taken from the Wande Database. The annual estimate of carbon dioxide emissions (CO_2_) is based on the third reference method for energy performance consumption given in the 2006 edition of the IPCC “Guidelines for National Greenhouse Gas Emission Inventory” (IPCC, 2006). Eight types of fuel are used to estimate carbon emissions: raw coal, coke, crude oil, fuel oil, gasoline, kerosene, diesel oil, and natural gas. The formula for the calculation is shown in formula (7):

(7) $$CO_{it}=\sum_{j=1}^{9}Y_{it,j}=\sum_{j=1}^{9}E_{it,j}\times LCV_{j}\times CC_{j}\times COF_{j}\times \frac{44}{12}$$

In formula (7), *CO_it_* represents the total carbon emissions of province/region *i* in year *t*; *j* indicates the eight types of fuel; *E_it,j_* represents the energy consumption of fuel type *j* in province *i* in year *t*; *LCV_j_* represents the lower calorific value of fuel type *j*; *CC_j_* represents the carbon content of fuel type *j*; *COF_j_* represents the carbon oxidation factor for fuel type *j*, and here the default emission factor in the IPCC report is used, which treats all fuel consumption as complete combustion (oxidation rate = 1); and 44/12 is the ratio of the molecular weight of carbon dioxide to that of carbon (the periodic table of the elements, Dmitri Mendeleev, 1869; International Union of Pure and Applied Chemistry, 2018). The data on energy consumption of various provinces and districts are taken from “China Energy Statistical Yearbook”, and the data on LCV, CC, and COF are taken from IPCC (2006).

With respect to financial support indicators, reference is made to the research literature, and the level of financial development in each province is measured from the three perspectives of financial structure, financial scale, and financial efficiency. The data come from China Statistical Yearbook and Wind Database.

Structural variables that reflect financial support are denoted FJG below. Bai Qinxian (1989) [62] pointed out in the “Comparative Banking Studies” that the financial structure can be described in terms of the number of finance-related factors, the relationship between them, and their proportional weightings. The changes in the financial structure represent changes in the level of financial development. The core of the financial structure is the financing structure. Therefore, the ratio of the loan balance at the end of the financial institution to the total financial assets is used to represent the financial structure variable.

Scale variables that reflect financial support are denoted FGM below. To measure a country’s level of financial development, Goldsmith (1969) [63] proposed a financially relevant ratio indicator (Financial Interrelations Ratio, FIR). FIR is the ratio of total financial activity over a certain period to total economic activity over that same period. FIR is directly proportional to the currency ratio, nonfinance-related ratio, capital formation ratio, external financing ratio, financial institution, new issuance ratio, and the financial asset price fluctuation and multiplier, and inversely proportional to the real income growth rate, price increase rate, and average capital output ratio. FIR measures the trend in financial development. This article uses a simplified FIR indicator to measure the financial level of each province, that is, FGM = total financial assets/GDP. From the perspective of asset liquidity, financial assets can be divided into currencies, bonds, stocks, and premium income. In view of this, the sum of total capital used by financial institutions, the market value of stock circulation, bond balances and premium balances is used in this paper.

The efficiency variable that reflects financial support is denoted FXL below. The basic function of the financial system is to guide the conversion of savings to investment, that is, to minimize the country’s idle funds and to optimize the allocation of investment. The efficiency of the financial system is embodied in the efficiency of the circulation of funds, that is, the ability of financial institutions to achieve optimal allocation of resources as a financial intermediary. Financial efficiency is expressed as the ratio of the total amount of loans of financial institutions to the total deposits of financial institutions, which can effectively measure the capital allocation ability of financial intermediaries to convert deposits into loans. The larger the value of this indicator, the stronger is the fund allocation ability of financial intermediaries and the higher is the financial efficiency.

The technological progress variable is measured as the total internal expenditure on R&D activities (denoted RD) and per capita patent applications (denoted PAT). RD indicates expenditures for R&D activities, and PAT indicates expenditures for R&D activities. The internal R&D expenditures come from the “China Statistical Yearbook”. Per capita patent applications are represented by the ratio of the number of authorized patents to the regional population at the end of the year. The units are tens of thousands, and the number of patent grants is derived from the “Statistical Yearbook of China’s Science and Technology”. The end-of-year demographic data for each province come from the Wind Database. The definition and measurement of the variables are shown in Table 1.

## 4. Empirical Analysis

### 4.1. Provincial Spatial Autocorrelation Test of Carbon Emissions

Before establishing the spatial econometric model, it is first necessary to test whether the interpreted variables are actually correlated spatially. The commonly used tests are Moran’s I index and the robust LM test.

The statistics of the global Moran’s I index can be expressed as

(8) $$I=\frac{n}{S_{0}}\frac{\sum_{i=1}^{n}\sum_{j=1}^{n}w_{i,j}z_{i}z_{j}}{\sum_{i=1}^{n}z_{i}^{2}}$$

where *z_i_* is the deviation of the attribute of element *i* from its mean value, $\left(x_{i}-\bar{X}\right)$; $w_{i,j}$ is the spatial weight between elements *i* and *j*; *n* is equal to the total number of elements; and *S*_0_ is the set of all spatial weights:

(9) $$S_{0}=\sum_{i=1}^{n}\sum_{j=1}^{n}w_{i,j}$$

The statistical *Z_I_* score is calculated as follows

(10) $$Z_{I}=\frac{I-E\left[I\right]}{\sqrt{V\left[I\right]}}$$

Among them

(11) E[I]=−1(n−1)

(12) $$V\left[I\right]=E\left[I^{2}\right]-E{\left[I\right]}^{2}$$

**Figure 2:**
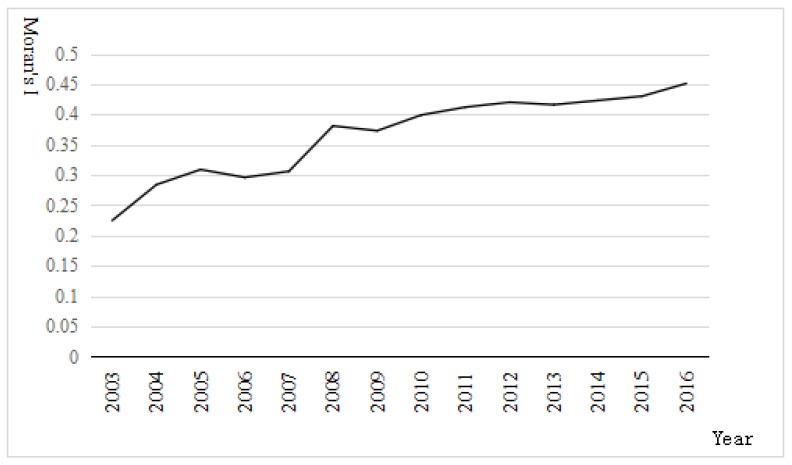
Global Moran’s I index of carbon intensity in China.

Moran’s *I* > 0 indicates spatial positive correlation, and the larger the value, the greater is the spatial correlation; Moran’s *I* < 0 indicates negative spatial correlation, and the larger the value, the larger the spatial difference; if Moran’s *I* = 0 and the space is random. Based on the spatially adjacent weight matrix, this paper uses stata software to perform the global Moran’s *I* index test on the raw data of the interpreted variables from 2003 to 2016, as shown in Figure 2. It can be seen that China’s interprovincial carbon intensity declined in every year over the period 2003–2016, and Moran’s I index of carbon intensity increases in volatility, and is above 0.225 every year, both surpassing the 5% level of significance. This shows that the spatial correlation of carbon intensity in various provinces in China is becoming increasingly strong. This is consistent with the findings of Honggang Cao, Kai Chen and Xin Tong (2015) [64], and Honglei Niu (2019) [65], which show a strong positive correlation between the total carbon emissions of the provinces.

Table 2 presents the residual test results based on the traditional hybrid panel data model with a spatial fixed effect, a time-fixed effect, and a bidirectional fixed effect. When using the traditional LM test, it can be seen that, with significance levels set to 1% and 10%, the null hypothesis that the interpreted variables and explanatory variables in the model have no spatial lag is rejected. When robust LM tests (Robust-LM) are used, the assumption that there is no spatial autocorrelation error term is also rejected. Combined with the calculated Moran’s I index, the carbon intensity among the provinces has a strong spatial correlation and it is necessary to establish a spatial econometric model to analyze each variable.

In order to determine spatial fixed effects and time-fixed effects, the likelihood ratio (LR) test was performed and the original hypotheses were rejected. The test results were estimated 246.4011, *ρ* = 0.0031 and estimated 512.0288, *ρ* = 0.000. This shows that the model can be extended to a model with a fixed spatial effect and a fixed time effect, that is, the model has a bidirectional fixed effect. According to traditional LM and robust LM test results, LMlag is significantly better than LMeroor in the LM test, Robust-LMlag is also better than Robust-LMerror, and it can be judged that a bidirectional fixed-effect spatial lag model should be adopted. LeSage and Pace (2009) [66] suggested that the results obtained by estimating model parameters from Wald statistics or LR statistics should be tested to verify whether the null hypotheses H0:θ = 0 and H0: θ+δβ=0 are supported, to determine whether the spatial Durbin model can be reduced to spatial lag and spatial error models. If the original hypothesis cannot be supported at the same time, it means that the spatial Durbin model cannot be reduced to a spatial lag model or a spatial error model. The established model should include the spatial lag of the dependent variable and the spatial lag of the independent variable to examine the spatial interaction between variables.

### 4.2. Analysis of the Spatial Durbin Model Estimation Results

In order to estimate the relationship between financial support, technological progress, and carbon intensity, this paper carries out estimation and significance tests under the adjacent weight matrix, economic weight matrix, and geographical weight matrix, as shown in Table 3. First, according to the Wald statistics and LR statistics, it can be judged whether the space panel Durbin model can be reduced to a spatial lag model or a spatial error model. The results of the Durbin model under the three matrices all pass the 1% level of significance, which shows that compared with the spatial error model and the spatial lag model, the spatial Durbin model is more suitable for the study of this problem. Then, according to the results of the Hausman test (Hausman estimate of adjacency matrix = 38.4227, y = 0.076523; economic matrix = 29.6850, y = 0.008707; geographical matrix = 48.0185, y = 0.045003), the econometric model of the spatial panel data should adopt the bidirectional fixed effect model. Thus, the spatial Durbin model under the bidirectional fixed effect was selected.

From the spatial Durbin model estimation of the bidirectional fixed effect in Table 3, the lag correlation coefficient, W*lnY, of the three explanatory variables under the matrix is positive at the 5% level of significance, which indicates that there is indeed a positive spatial spillover effect between a region’s carbon intensity and that of its neighboring regions. The geographical proximity of regions and the mobility of products, services, technologies, and funds between regions have promoted the transfer of carbon emissions among regions. From the perspective of economic development, the increase in regional demand not only stimulates economic growth in the region, but also moves resources through the region, which has a positive effect on the economy of other regions. However, economic production is generally accompanied by an increase in carbon emissions, so that the economic development of one region is driven by other regions, accompanied by the carbon emissions generated during the process of undertaking economic development. With greater inter-regional cooperation, technology sharing, and financial exchanges, this influence will continue to spread. One region will bear some of the carbon emission pressures for other regions and further expand the degree of interprovincial carbon emissions in China (Yao Liang and Liu Jingru, 2010 [67]; Lianqing Peng, 2008 [68]).

Comparing the estimation results of the three types of weight matrix, there are spatial spillover effects of the spatial lag term of the dependent variable and the spatial interaction term of the independent variable, which are as follows.

(1) There are certain differences in the size and direction of the coefficient of influence of the three financial support variables. Excluding the regression coefficients of lnFXL under the economic weight matrix, the coefficients on the three matrix financial support variables are statistically significant: lnFJG and lnFGM are significantly positive and the lnFXL regression coefficients of the adjacent weight matrix and geographical weight matrix are significantly negative. This indicates that there is a significant positive correlation between financial structure and financial scale and carbon intensity within the sample range, and an improvement in financial efficiency reduces regional carbon intensity. This is in line with the results of the study by Gu Mi (2017) [69], who found that financial efficiency restrains carbon emissions and financial scale promotes carbon emission intensity.

(2) The elastic coefficients of the variables lnRD and lnPAT, representing technological progress under the three types of weight matrix, are significantly negative, indicating that the internal expenditure on R&D and the number of patent applications per capita are both beneficial to inhibiting regional carbon intensity. Comparing the estimation results of the three types of weight matrix, there are differences in the coefficient of influence of the two indicators of technological progress, and the negative impact of the number of patent applications per capita on carbon intensity is relatively weak. This may be because of the fact that the patent application variable has a certain time lag in its impact on carbon intensity. Patented inventions will proliferate only after a period of time, and it is difficult to suppress carbon intensity in the short term. Likewise, Wenxing Ding (2018) [70] believes that internal expenditure on R&D activities can improve the production efficiency of enterprises, promote research and development and the use of “new” (green) energy, and play a role in curbing carbon emissions.

(3) Further analysis of the estimated coefficient and significance level of the spatially lagged terms of financial support and technological progress reveals that the spatially lagging term estimating coefficients of the technological progress variables lnRD and lnPAT under the three types of weight matrix are significant (at least at a confidence level of 10%), which indicates that with further technological development, its spatial negative external effects on carbon intensity gradually increase and carbon intensity in adjacent areas is reduced. The coefficient of spatial lag for financial support differs across the three types of weight matrix. The lagged coefficient of financial structure is significantly positive, indicating a significant spatial spillover effect of financial structure. The financial structure of neighboring provinces shows an agglomeration effect on the growth in carbon intensity. The spatial lag coefficient of financial scale is significantly negative only under the economic weight matrix, while the variable of financial efficiency is significantly negative under both the economic weight matrix and the geographical weight matrix, which indicates that financial scale and financial efficiency can lead to a decline in the carbon intensity of neighboring provinces, and the factors within the province and the factors affecting the neighboring provinces jointly drive the changes in China’s provincial carbon intensity.

LeSage and Pace (2009) [66] proposed that partial differentials can explain whether the variation in variables in different model settings has a spatial spillover effect on neighboring regions, and they believe that the spatial regression model may have misinterpreted the effect of spatial regression on other regions in the spatial regression model. Further, the influence of financial support and technological progress on carbon intensity can be decomposed into direct, indirect, and total effects under the spatial Durbin model to test the effects of explanatory variables on interpreted variables in the region of interest, other regions, and all regions of the country by spatial interactions, as shown in Table 4. The decomposition results of the three spatial weight matrix spillover effects are specifically analyzed as follows:

Direct effects of the spatial Durbin model. Under the three types of weight matrix, except for the fact that financial efficiency is not significant under the geographical spatial weight matrix, the regression coefficients of other financial support variables are significantly negative, indicating that these factors have a negative impact on the carbon intensity of the region. In addition, among the coefficient values under the three spatial weight conditions, the absolute value of the financial scale factor is the largest, followed by that for the financial structure, which indicates that financial scale has the greatest effect on the suppression of carbon intensity in the region. With the rapid development of the financial industry in the region, the construction of a carbon financial system has been accelerated, which will help guide the accumulation of investments in green development. Gu Mi (2017) [69] believes that greater financial scale can reduce the cost of corporate finance, and thereby increase investment in technological innovation and the industrial upgrading of enterprises; in turn, this will reduce the carbon emission intensity of the region. The impact coefficients of R&D expenditures on the carbon intensity of the region are 0.069459, −0.168532, and 0.163037, respectively; all of them are significant at the 5% level and are significantly negative only under the economic weight matrix, indicating that, after considering the economic factors, R&D expenditures restrain carbon intensity. As technological progress is closely related to financial support and economic development, with the spatial distribution of economic regions being centralized, a coordinated and sustainable development of the economy has been achieved with respect for the environment and the exploitation of natural resources. The per capita number of patent applications has a significant negative impact on carbon intensity under the three spatial weight matrixes.

Indirect effects of the spatial Durbin model. The regional spillover effect of financial structure and financial scale is negative under the economic weight matrix, but it is not statistically significant. Only the financial structure variable has a significant positive effect on the carbon intensity of other regions under the geographical weight matrix, indicating that due to the large regional differences in China, the carbon emission reduction targets and approaches are not the same in all regions and the role of the increase in total financial assets in suppressing carbon intensity has not been fully realized. The financial efficiency variable has a significant negative effect on the carbon intensity of other regions under the three weight matrix conditions, which also reflects the fact that financial credits help to promote energy conservation and emission reduction. In the lnRD and lnPAT spillover effects that represent technological progress variables, most of the coefficients of the explanatory variables are significantly negative; in particular, the coefficients of lnRD are smaller than those of the direct effects, indicating that the intra-area spillover effect of technological progress is stronger than the inter-regional spillover effect, and technological progress has an effective role in reducing carbon intensity. Therefore, at the policy level, it would be desirable to promote technological research and development.

Comparing the results of the spatial Durbin model under the three types of weight matrix, the estimation result under the economic weight matrix is more objective and comprehensive. The degree of marketization of provinces and cities with higher levels of economic development is higher, and economic development can be combined with a low-carbon economy, which in turn is conducive to improving technological development, as well as the coordinated development of technological advancement, financial support, and a low-carbon economy.

## 5. Conclusions

This paper empirically analyzes the direction and degree of the impact of financial support and technological progress on carbon intensity, in spatial terms. It does so by establishing a spatial Durbin model, based on the partial differential method of spatial regression modeling, and taking into account the influence of different spatial weights on spatial effects combined with China’s 2003–2016 provincial panel data. The results show that there is a spatial correlation in China’s carbon intensity and that the degree of spatial agglomeration is continuously enhanced. Carbon intensity exhibits a significant spatial spillover effect under different spatial weights, indicating that a province’s carbon intensity is affected not only by its own characteristics, but also by carbon emissions from geographically adjacent and economically similar provinces and regions. This spillover effect under the weight of economic space is stronger than under other spatial weights. The variables that represent financial support have different impacts on carbon intensity. Financial structure, financial scale, and financial efficiency have significant effects on reducing carbon intensity in the region, while financial scale has no significant spillover effect on carbon intensity in space. Areas with higher financial efficiency reduce the intensity of local carbon emissions, but also radiate this to the surrounding areas, suppressing the carbon intensity of neighboring regions, and thereby promoting the reduction of carbon emissions across the country. Combined with the results of Hong Jiang (2018) [26], financial scale and financial efficiency are found to significantly increase energy efficiency in the region and surrounding areas. It can be seen that financial efficiency can inhibit carbon emissions by improving energy efficiency, but financial scale has less impact on curbing carbon emissions in surrounding areas by improving energy efficiency. The empirical results on internal spending on R&D activities and on per capita patent applications (representing technological progress) show that most of the coefficients of explanatory variables are significantly negative. Compared with the coefficients of direct effects, the extent of spillover effects of technological progress on reducing carbon intensity is significantly less, and the intraregional spillover effect of technological progress is greater than the inter-regional spillover effect. Although the patent application variable has a positive effect on the suppression of carbon intensity, it has a certain time lag.

The following recommendations are made in response to the above conclusions. Firstly, China should increase cooperation among provinces, for example, with infrastructure sharing and information and knowledge exchange. This will allow the more poorly performing provinces to learn from and absorb the reform results of high-quality low-carbon provinces and cities. Each province needs to develop a low-carbon development strategy that suits itself, and should encourage industrial cities to carry out technological innovation. This will give full play to the demonstration and diffusion effects of high-quality low-carbon provinces and cities. Secondly, China should consider the complex effects of industrial structural factors on reducing carbon intensity and the impact on different regions when formulating carbon emission policies. It should continue to develop a low-carbon economy, paying attention to carbon emissions in the industrial sector, which accounts for the largest proportion of carbon emissions. The optimization and upgrading of industrial structure is one of the important ways to reduce carbon emissions. Financial tools can be used to further promote the development of a low-carbon economy and enhance the utilization efficiency of financial resources and energy. Vigorously support is warranted to increase the level of financial development as financial development is an important means to achieve coordinated and environmentally sustainable economic and environmental development. Under the premise of balancing economic development and environmental protection, China should optimize its financing structure, actively guide the effective integration of credit policies and low-carbon economic industrial policies, and give full play to the spillover effects of financial efficiency on reducing carbon intensity. Thirdly, further increase investment in research and development funding and investment in innovation and promotion of low-carbon technologies should be further increased. Low-carbon technologies such as emission reduction technologies and energy technologies are key to reducing the intensity of carbon emissions, and technological advancement is inseparable from financial support. Finally, attention should be given to the technological progress of the provincial and interprovincial spillover effects of technological progress on reductions in carbon emissions. China should effectively promote scientific and technological research and development results, give full play to the use of technological achievements that have value in suppressing carbon emission intensity, and actively promote the development of a low-carbon economy.

## Figures and Tables

**Table 1:** Definition and measurement of variables.

Variables	Definition and Measurement
Carbon intensity Y	Y = CO_2_/GDP, in tons/ten thousand yuan.
Scale variables that reflect financial support FGM	FGM = Total financial assets/GDP = (Total financial resources used by financial institutions + Stock market value + Bond balance + Premium balance)/GDP
Structural variables that reflect financial support FJG	FJG = Financial institution loan balance at the end of the year/Total financial assets = Financial institution loan balance at the end of the year/(Total financial resources used by financial institutions + Stock market value + Bond balance + Premium balance)
Efficiency variable that reflects financial support FXL	FXL = Total loans of financial institutions/Total financial institutions deposits
R&D expenditures internal expenditures RD	Measured by the internal expenditures of R&D expenditures in various regions; the unit is 100 million yuan.
Per capita patent applications PAT	PAT = Patent authorization number/Year-end population; the unit for which is 10,000 people.
Adjacency weight matrix $W_{ij}^{1}$	$W_{ij}^{1}=\left\{ \begin{array}{l} 1,\mathrm{when}\;i\;\mathrm{and}\;j\;\mathrm{are}\;\mathrm{adjacent}\\ 0,\mathrm{Other}\;\mathrm{situations} \end{array}\right.$, that is, if two regions are geographically adjacent, let $W_{ij}^{1}=1$, otherwise 0.
Geographic weight matrix $W_{ij}^{2}$	$W_{ij}^{2}=\left\{ \begin{array}{l} \frac{1}{D_{ij}^{2}},\mathrm{when}\;i\;\mathrm{and}\;j\;\mathrm{are}\;\mathrm{adjacent}\\ 0,\mathrm{Other}\;\mathrm{situations} \end{array}\right.$, *D_ij_* is the distance between the two capital cities calculated by latitude and longitude.
Economic weight matrix $W_{ij}^{3}$	$W_{ij}^{3}=\left\{ \begin{array}{l} \frac{1}{|GDP_{i}-GDP_{j}|},\mathrm{when}\;i\;\mathrm{and}\;j\;\mathrm{are}\;\mathrm{adjacent}\\ 0,\mathrm{Other}\;\mathrm{situations} \end{array}\right.$, GDP as per region GDP per capita during the sample period

**Table 2:** Estimation results of traditional hybrid panel data model.

Variable	Spatial Fixed Effect	Timed Fixed Effect	Two-Way Fixed Effect
lnFJG	0.584253 *** (4.106919)	0.240464 *** (4.033609)	−0.015657 *** (9.649219)
lnFGM	0.639430 *** (6.340458)	−0.030902 (0.928114)	−0.057254 *** (5.085647)
lnFXL	0.029533 (0.238726)	−0.213918 *** (−4.530700)	0.250308 *** (−4.509987)
lnRD	0.160020 *** (−5.350509)	−0.058099 *** (−3.242875)	−0.145259 ** (−2.382171)
lnPAT	−0.056001 ** (−2.321551)	−0.272632 *** (−2.716159)	−0.212585 *** (−2.716159)
R^2^	0.1914	0.3983	0.8030
Log-likelihood	304.6192	−248.4110	201.5277
LMlag	3.7554 *	95.1616 ***	18.3844 ***
Robust-LMlag	1.8364	22.6243 ***	5.9763 **
LMerror	2.6604	72.5769 ***	14.3132 *
Robust-LMerror	0.7414	0.0396	1.9051

Note: The *t*-statistics are in parentheses, * is significant at 10% confidence level, ** is significant at 5% confidence level, and *** is significant at 1% confidence level, the same below.

**Table 3:** Spatial Durbin model estimation and test.

Variables	Two-Way Fixed Effect			Random Effect		
	Adjacency Matrix	Economic Matrix	Geographic Matrix	Adjacency Matrix	Economic Matrix	Geographic Matrix
lnFJG	0.425188 ***	0.566384 ***	0.603039 ***	0.245802 ***	0.682680 ***	0.444862 ***
lnFGM	0.419447 ***	0.623021 ***	0.613533 ***	0.201089 **	0.730343 ***	0.481504 ***
lnFXL	−0.013464 *	0.024588	−0.047724 **	0.057773 *	−0.050647 *	0.194269 *
lnRD	−0.14500 ***	−0.172148 ***	−0.169546 ***	0.057233 ***	0.140653 ***	0.128634 ***
lnPAT	−0.017683 **	−0.044200 *	−0.035167 ***	−0.090255 **	−0.121580 ***	−0.051648 *
W*lnFJG	0.039356 *	0.303015 *	1.080740 ***	−0.016419 *	−0.551633 ***	0.295951
W*lnFGM	0.148659	−0.344371 *	0.282024	−0.055532 *	−0.617813 ***	−0.316510 **
W*lnFXL	0.134168	−0.196592 **	−1.245640 ***	−0.193713 **	0.097805	−0.625544 ***
W*lnRD	−0.115651 **	−0.081394 *	−0.187231 *	0.020980 *	−0.045078 ***	0.024865
W*lnPAT	−0.042882 **	−0.141555 **	−0.130469 *	−0.134885 ***	−0.166242 ***	−0.217064 ***
W*lnY	0.304158 ***	0.586095 ***	0.276900 ***	0.602980 ***	0.505000 ***	0.566995 ***
teta				0.072218	0.054061	0.057969
Wald_spatial_lag	21.5664 ***	19.0941 ***	16.9135 ***	12.9903 ***	13.8764 ***	20.0609 ***
LR_spatial_lag	23.1102 ***	22.0631 ***	24.3116 ***			
Wald_spatial_error	28.4327 ***	21.8123 ***	22.6681 ***	16.2104 ***	12.0887 ***	16.6862 ***
LR_spatial_error	26.8217 ***	18.0258 ***	20.766			

Note: teta is a random effect; adding * indicates that the test results are significant at 10% confidence level, ** indicates that the test results are significant at 5% confidence level and *** indicates that the test results are significant at 1% confidence level.

**Table 4:** Direct effect, indirect effect, and total effect of the spatial Durbin model.

Variables	Effect	Adjacency Weight Matrix	Economic Weight Matrix	Geographic Weight Matrix
lnFJG	Direct Effect	−0.270553 ** (2.572870)	−0.567431 *** (4.776008)	−0.667972 *** (5.532768)
	Indirect Effect	0.314399 (0.934393)	−0.229892 (−0.792254)	1.720065 *** (3.258294)
	Total Effect	0.043846 * (1.775649)	−0.797323 (1.079492)	1.052093 *** (4.241222)
lnFGM	Direct Effect	−0.212548 ** (2.146843)	−0.622376 *** (5.445147)	−0.638185 *** (5.449312)
	Indirect Effect	0.252683 (0.588840)	−0.269054 (0.588840)	0.924663 (1.453559)
	Total Effect	0.040135 * (1.859323)	−0.89143 (1.198610)	0.286478 *** (2.774347)
lnFXL	Direct Effect	−0.024885 * (1.906190)	−0.023065 * (−1.870949)	0.111247 (−0.777147)
	Indirect Effect	−0.369290 *** (−2.736349)	−0.241266 ** (2.359365)	−1.739281 *** (−2.961039)
	Total Effect	−0.394175 * (−1.961541)	−0.264331 (0.638459)	−1.628034 *** (−2.797616)
lnRD	Direct Effect	0.069459 ** (2.290024)	−0.168532 *** (4.304425)	0.163037 *** (4.028740)
	Indirect Effect	−0.130082 ** (2.304254)	−0.057522 (−0.452338)	−0.186815 (−1.286994)
	Total Effect	−0.060623 *** (3.396772)	−0.226054 (0.838887)	−0.023778 (0.838887)
lnPAT	Direct Effect	−0.440598 *** (−8.745832)	−0.157121 ** (2.012049)	−0.191621 * (−1.928890)
	Indirect Effect	−0.128854 *** (−5.546450)	−0.040166 * (−1.935555)	−0.042469 * (−1.99295)
	Total Effect	−0.569452 *** (−10.708107)	−0.197287 (1.255859)	−0.234090 ** (−2.322743)

Note: adding * indicates that the test results are significant at 10% confidence level, ** indicates that the test results are significant at 5% confidence level and *** indicates that the test results are significant at 1% confidence level.
